# Discrepancy between two invasive blood pressure measurements in patients receiving intra-aortic balloon pump therapy

**DOI:** 10.1186/s12872-023-03479-2

**Published:** 2023-09-09

**Authors:** Lijuan Lu, Shiyi Zhang, Yu Zhang, Xiaoyan Zhao

**Affiliations:** Nursing Department, Tongji Hospital, Tongji Medical College, Huazhong University of Science and Technology, No. 1095, Jiefang Avenue, Wuhan, 430000 Hubei Province China

**Keywords:** Cardiogenic shock, Coronary care unit, Invasive central aortic pressure, Invasive peripheral arterial pressure, Intra-aortic balloon pump

## Abstract

**Background:**

Hemodynamic monitoring is imperative for patients with cardiogenic shock undergoing Intra-aortic Balloon Pump (IABP) therapy. Blood pressure monitoring encompasses non-invasive, invasive peripheral arterial pressure (IPAP), and invasive central aortic pressure (ICAP) methods. However, marked disparities exist between IPAP and ICAP. This study examined the discrepancies between IPAP and ICAP and their clinical significance.

**Methods:**

A retrospective analysis was conducted on cardiogenic shock patients who underwent IABP therapy and were admitted to the Coronary Care Unit (CCU) of a tertiary hospital in China from March 2017 to November 2022. The Bland–Altman plot illustrated the discrepancy between IPAP and ICAP. A clinically significant difference between ICAP and IPAP measurements was defined as ≥ 10 mmHg, which could necessitate alterations in blood pressure management according to current guidelines that recommend maintaining a mean arterial pressure (MAP) ≥ 70 mmHg.

**Results:**

In total, 162 patients were included in the final analysis. In patients without vasopressors, the difference between ICAP and IPAP was 5.73 mmHg (95% limits of agreement [LOA], -16.98 to 28.44), whereas, in patients with vasopressors, it was 4.36 mmHg (95% LOA, -17.31 to 26.03). ICAP measurements exceeded IPAP in patients undergoing IABP therapy. However, the difference was not statistically significant between the two groups. Multivariate logistic regression revealed that higher serum lactate levels (Odds ratio [OR], 1.14; 95% confidence interval [CI], 1.03–1.27; *p* = 0.013) and age ≥ 60 years (OR, 13.20; 95% CI, 1.50–115.51; *p* = 0.020) were associated with an increased likelihood of a clinically significant MAP discrepancy. Conversely, a history of coronary heart disease was associated with a decreased likelihood (OR, 0.34; 95% CI, 0.13–0.90; *p* = 0.031).

**Conclusions:**

Notable discrepancies between ICAP and IPAP measurements exist in cardiogenic shock patients undergoing IABP therapy. ICAP exceeds IPAP, and factors such as age ≥ 60 years, elevated lactic acid levels, and absence of coronary heart disease contribute to this discrepancy. Enhanced vigilance is warranted for these patients, and the consideration of peripheral invasive monitoring in conjunction with IABP therapy is advised.

**Supplementary Information:**

The online version contains supplementary material available at 10.1186/s12872-023-03479-2.

## Introduction

Cardiogenic shock (CS) constitutes a critical condition characterized by the heart’s inadequate contractility, which compromises the maintenance of sufficient cardiac output due to insufficient myocardial oxygenation and elevated cardiac workload [1]. Consequently, CS can lead to multi-organ failure [2] and significant mortality rates [3]. Its prevalence varies depending on the underlying cause (e.g., the rate is approximately 3% ~ 13% among hospitalized patients with acute myocardial infarction) [4]. Managing CS is a pivotal aspect of care within coronary care units (CCUs). Since its inception in 1968, intra-aortic balloon counterpulsation (IABP) has emerged as the predominant mechanical support modality in the management of CS [5].

Globally, over 4.5 million cases have been managed using IABP, including more than 300,000 in China alone, and the usage rate is witnessing an annual escalation between 13 and 20%. IABP entails the deployment of a balloon catheter within the descending aorta, which is regulated by an external device. During diastole, balloon inflation elevates diastolic pressure, which fosters improved coronary circulation. Conversely, deflating the balloon prior to systole diminishes the resistance against systolic output, consequently enhancing myocardial oxygenation and reducing oxygen consumption by mitigating cardiac workload.

Efficient management of IABP necessitates the monitoring of numerous parameters, among which the assessment of hemodynamic status is of utmost importance [6–9]. Healthcare professionals involved in IABP management are required to engage in ongoing assessment of hemodynamic status, which entails the analysis of arterial pressure waveforms. However, it is imperative to acknowledge that different devices may yield varying interpretations of these waveforms. Typically, bedside monitors identify the peak of the arterial pressure waveform as the systolic blood pressure (SBP) and the nadir as the diastolic blood pressure (DBP). During IABP therapy, the balloon augmentation pressure, which occurs during diastole, emerges as the peak of the waveform, potentially being mistaken for SBP. Consequently, mean arterial pressure (MAP) may also be subject to distortions depending on the monitor's algorithm. Moreover, the conventional arithmetic calculation for MAP (i.e., (SBP + 2*DBP)/3) does not accurately represent the effect of balloon-assisted diastolic augmentation [10].

Studies indicate that SBP and DBP measurements can exhibit a discrepancy of 10–15 mmHg when obtained from central versus peripheral sites. This variance arises as the pulse travels through the arterial system, leading to increased SBP and potentially decreased DBP due to the reflection of the pressure wave. For instance, peripheral SBP, as measured from the radial artery, is usually higher than aortic SBP, a phenomenon termed the physiological amplification of central pulse pressure [11]. However, MAP is observed to remain stable across different arterial sites [12]. A minimum MAP of 70 mm Hg is recommended to mitigate hypoperfusion [13]. Nevertheless, variations in MAP have been reported among critically ill patients undergoing cardiac surgery [14, 15], cardiopulmonary bypass [16–18], liver transplantation [19, 20], or experiencing septic shock [21, 22]. Notably, the administration of vasopressors is associated with an increased likelihood of clinically significant discrepancies in blood pressure measurements.

Accurate blood pressure monitoring is indispensable for CS patients undergoing IABP therapy, as it imparts critical insights into the hemodynamic status and informs therapeutic decision- making [10]. Notably, the impact of blood pressure monitoring on patients undergoing IABP therapy has not been elucidated in prior research. The discordance between peripheral and central blood pressure measurements can culminate in imprecise evaluation of organ perfusion, tardy identification of hemodynamic compromise, and misjudgment of therapeutic outcomes. A prospective investigation involving 36 patients undergoing IABP revealed that non-invasive blood pressure (NIBP) tends to exaggerate readings in comparison to central arterial pressure (CAP), resulting in premature cessation of vasopressors and consequent under-perfusion in vulnerable patients. Specifically, non-invasive SBP exhibited a substantial deviation of + 19.8 mmHg, which is clinically untenable [23]. Some authorities posit that MAP as displayed on the IABP console is the most reliable representation of central aortic pressure. However, the evidence for employing IABP-derived blood pressure (CAP) is lacking, necessitating further exploration. Currently, blood pressure management relies on peripheral measurements, and the optimal method of blood pressure monitoring in this patient cohort remains unclear. Though invasive peripheral arterial pressure (IPAP) is routinely documented, there is an absence of literature examining the association between IPAP and invasive central aortic pressure (ICAP) in this population. Critically, preceding studies did not probe the influence of ICAP monitoring via IABP on clinical management (e.g., the necessity of vasopressors or other interventions) relative to peripheral blood pressure monitoring.

In our study, we aimed to investigate the discrepancy between peripheral blood pressure and CAP in a large patient population undergoing IABP therapy with vasopressors, compared to a control cohort treated with IABP without vasopressors. We hypothesized that the administration of vasopressors might be associated with amplified discrepancies between IPAP and ICAP measurements, potentially impacting clinical management strategies for patients receiving IABP.

## Methods

### Ethical statement

This study was approved by the ethics committee of Tongji Hospital, Tongji Medical College of Huazhong University of Science and Technology(TJ-IRB20220955). Informed consent was waived due to the retrospective nature of the study and the utilization of anonymized data from electronic medical records.

### Study design and setting

This was a single-center, retrospective study conducted at the CCU of a tertiary-level general hospital in China, which predominantly serves patients with severe coronary heart disease (CHD), severe arrhythmia, cardiac insufficiency, cardiomyopathy, fulminant myocarditis and other severe cardiac ailments. Upon admission to the CCU, attending physicians promptly initiate electrocardiogram (ECG) monitoring. Typically, patients necessitating regular arterial blood gas analyses or intensive hemodynamic monitoring are subjected to invasive arterial pressure monitoring. Furthermore, as stipulated by the CCU’s clinical protocol, critically ill patients must have blood pressure measurements and vital sign documented at a minimum hourly frequency.

### Participants

The study population comprised patients admitted between March 2017 and November 2022, who underwent arterial blood pressure monitoring. Inclusion criteria entailed: (1) age over 18 years, (2) a confirmed diagnosis of cardiogenic shock, and (3) treatment with IABP with a counterpulsation ratio of 1:1, ensuring the equipment functioned optimally. Exclusion criteria encompassed patients diagnosed with hypertensive emergencies (e.g., acute aortic diseases, spontaneous intracranial hemorrhage, ischemic stroke, eclampsia) [24], severe arrhythmias (e.g., atrial fibrillation, frequent premature ventricular contractions), or those undergoing extracorporeal membrane oxygenation (ECMO) treatment, as the management of these patients adhered to SBP recommendations set forth by existing guidelines. Given that twelve independent variables were included in the logistics regression analysis, and according to five to ten times the number of independent variables (while accounting for 20% potentially invalid data), the estimated sample size ranged from 75 ~ 150 [24]. The final sample consisted of 162 patients.

### Blood pressure monitoring for patients undergoing IABP

#### Invasive central aortic pressure (ICAP) monitoring

IABPs were percutaneously inserted through the femoral artery under fluoroscopic guidance by an interventional physician. Following IABP treatment in the catheterization room, patients were transferred to the CCU. The system was calibrated to zero at the level of the right atrium, along the midaxillary line, and then secured. Prior to data collection, the arterial pressure measurement device underwent a routine "square wave test". ICAP data were acquired from the IABP console (Maquet, Rastatt, Germany), with the most recent three measurements being selected post-successful implantation (if vasopressors were administered, three measurements post-initiation were recorded). The average of these three measurements was compared.

#### Invasive peripheral aortic pressure (IPAP) monitoring

IPAP monitoring was conducted using a multi-function monitor (Myrui, China) and a disposable pressure sensor set (Braun, Germany). Experienced nurses performed arterial punctures with a 20G arterial indwelling needle (BD, USA). It is imperative to ensure the absence of air bubbles in the pressure measurement system and to prevent the pipeline from twisting or folding. The system was calibrated to zero at the level of the right atrium, along the midaxillary line, and then secured. A "square wave test" of the arterial pressure measurement device was routinely performed before data acquisition. Exclusion criteria included: (1) an unsmooth catheter with incomplete needle sleeve insertion, and (2) more than two punctures in the same area or the presence of local hematomas at puncture and catheter placement sites. Blood pressure data were collected concurrently with the CAP measurements.

### Data collection and management

Data were extracted from electronic medical records. We first collected demographic data such as age, gender, diagnosis, past medical history, and body mass index. The laboratory information system provided serum lactate and left ventricular ejection fraction (LVEF) values, which were extracted from the first medical records at the time of admission. Clinical data included three measurements each of CAP and peripheral arterial pressure, heart rate, vasopressor administration, duration of IABP treatment, and arterial catheterization. Additionally, data regarding catheterization complications during hospitalization were extracted. Complications of peripheral arterial catheterization were defined as infection, bleeding at the puncture site, hematoma and aneurysm. IABP-related complications included limb ischemia, thrombosis, bleeding and hematoma at the puncture site, infection, etc. Details of the data extraction form see Supplemental file 1.

### Outcomes

The primary outcome of this study was an analysis of the discrepancies between ICAP and IPAP measurements. The secondary outcome concerned the clinical relevance of the discrepancies between ICAP and IPAP. Clinical relevance was defined by: a) a difference of 10 mmHg or more between the two measurements, and b) potential alterations in blood pressure management in accordance with current guidelines [13]. For example, guidelines recommend maintaining a MAP of 70 mmHg or higher. A patient with an ICAP of 70 mmHg and an IPAP of 58 mmHg would exhibit a clinically relevant discrepancy in MAP, necessitating the initiation of vasopressors or crystalloids to elevate MAP. Conversely, a patient with an ICAP of 82 mmHg and an IPAP of 70 mmHg would not display a clinically relevant discrepancy in MAP, as no intervention would be warranted given that both measurements showed a MAP of 70 mmHg or higher. The study also estimated the proportion of patients with a discrepancy of 10 mmHg or greater between ICAP and IPAP.

### Statistical analysis

Descriptive analyses were performed to present continuous variables as mean ± standard deviation (SD) or median with interquartile range (IQR), and categorical variables as percentages. The paired t-test was used to assess the difference between ICAP and IPAP within the same group. To evaluate differences between two groups (patients with or without vasopressor treatment), independent t-tests or Mann–Whitney U tests were employed. Categorical data comparisons were conducted using Chi square tests.

The Bland–Altman plot was used to graphically depict the discrepancies between ICAP and IPAP. Forward stepwise multivariable logistic regression was employed to ascertain if independent variables could predict the outcomes of interest. Various independent variables potentially associated with blood pressure were controlled in the regression analysis. Additionally, probit regressions were conducted to estimate the proportion of patients exhibiting the primary outcome per unit change in continuous variables. The fit of the multivariate logistic regression models was assessed using Hosmer-Leeshawn tests, with a *p*-value > 0.05 indicating a good fit.

Analyses were performed using Minitab version 19 (Minitab Corp, State College, Pennsylvania, USA) and GraphPad Prism 8.3 (GraphPad Software, USA). A two-tailed *p* value < 0.05 was deemed statistically significant.

## Results

### Patient characteristics

**Fig. 1:**
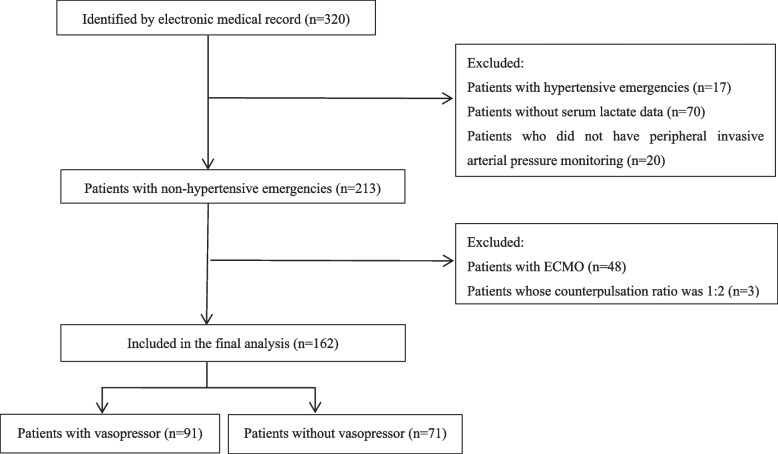
Patient selection diagram. ECMO, extracorporeal membrane oxygenation

A total of 320 patients who underwent IABP between March 2017 and November 2022 were initially enrolled in this study. Of these, 162 patients were included in the final analysis, comprising 71 patients who did not receive vasopressors and 91 patients who did (see Fig. 1).

The average age of the patients was 58 years, with a SD of 17 years, and 68% (110/162) of the participants were male. No statistically significant differences were observed in demographic characteristics such as age, gender, and body mass index, or in clinical factors associated with the location of the arterial catheter (see Table 1). However, statistically significant differences were noted in past medical history, utilization of mechanical ventilation, infarct culprit artery, serum lactate values, left ventricular ejection fraction, and hospital disposition (Table 1). The five most common diagnoses among the 162 patients were acute myocardial infarction (56%), myocarditis (20%), cardiogenic shock (7%), cardiomyopathy (5%), and heart failure (4%).

**Table 1:** Characteristics of patients who received arterial pressure monitoring in the Coronary Care Unit (*n* = 162)

Variables	All patients (*n* = 162)	Without vasopressor (*n* = 71)	With vasopressor (*n* = 91)	*p* value
Age, years (M, SD)	58 (17)	56 (18)	60 (16)	0.110
Gender, n (%)				0.199
Male	110 (68)	52 (73)	58 (64)	
Female	52 (32)	19 (27)	33 (36)	
BMI, (M, SD)	23.3 (3.7)	23.8 (3.4)	22.9 (3.9)	0.110
Diabetes, n (%)	47 (29)	23 (32)	24 (26)	0.402
HTN, n (%)	62 (38)	30 (42)	32 (35)	0.357
CHD, n (%)	107 (66)	53 (75)	54 (59)	0.041
Kidney disease, n (%)	48 (30)	12 (17)	34 (37)	0.001
PAD, n (%)	0 (0)	0 (0)	0 (0)	NA
Mechanical ventilation, n (%)				
None	92 (57)	51 (72)	42 (46)	< 0.001
IMV	47 (29)	9 (13)	38 (42)	
NIMV	22 (14)	11 (15)	11 (12)	
Location of arterial catheter, n (%)				
Radial	142 (88)	65 (92)	77 (85)	0.183
Brachial	20 (12)	6 (8)	14 (15)	
Infarct culprit artery, n (%)				
LAD	72 (44)	39 (55)	33 (36)	0.018
RCA	82 (50)	44 (62)	38 (42)	0.011
LCX	76 (47)	41 (58)	35 (38)	0.015
Diagnoses, n (%)				
Acute myocardial infarction	90 (56)	48 (68)	42 (46)	0.008
Myocarditis	32 (20)	14 (20)	18 (20)	0.868
Cardiogenic shock	11 (7)	2 (3)	9 (10)	0.144
Cardiomyopathy	8 (5)	3 (4)	5 (5)	0.996
Heart failure	7 (4)	1 (1)	6 (7)	0.222
Others	14 (8)	3 (4)	11 (12)	0.070
Serum lactate (mmol/L), median [IQR]	2.4 [1.6–3.8]	1.8 [1.3–2.6]	3.0 [2.0–5.0]	< 0.001
LVEF (M, SD)	37.3 (13.4)	39.3 (12.4)	35.7 (14.0)	0.090
Hospital disposition, n (%)				
Discharge home	140 (87)	67 (94)	73 (81)	0.009
Dead/Hospice	22 (13)	4 (6)	18 (19)	

*M* mean, *SD* standard deviation, *BMI* body mass index, *HTN* hypertension, *CHD* coronary heart disease, *PAD* peripheral arterial disease, *IMV* invasive mechanical ventilation, *NIMV* noninvasive mechanical ventilation, *LAD* left anterior descending, *RCA* right coronary artery, *LCX* left circumflex, *IQR* interquartile range, mmol/L millimoles per liter, *LVEF* left ventricular ejection fraction

### Bland–Altman consistency evaluation between ICAP and IPAP measurements

**Fig. 2:**
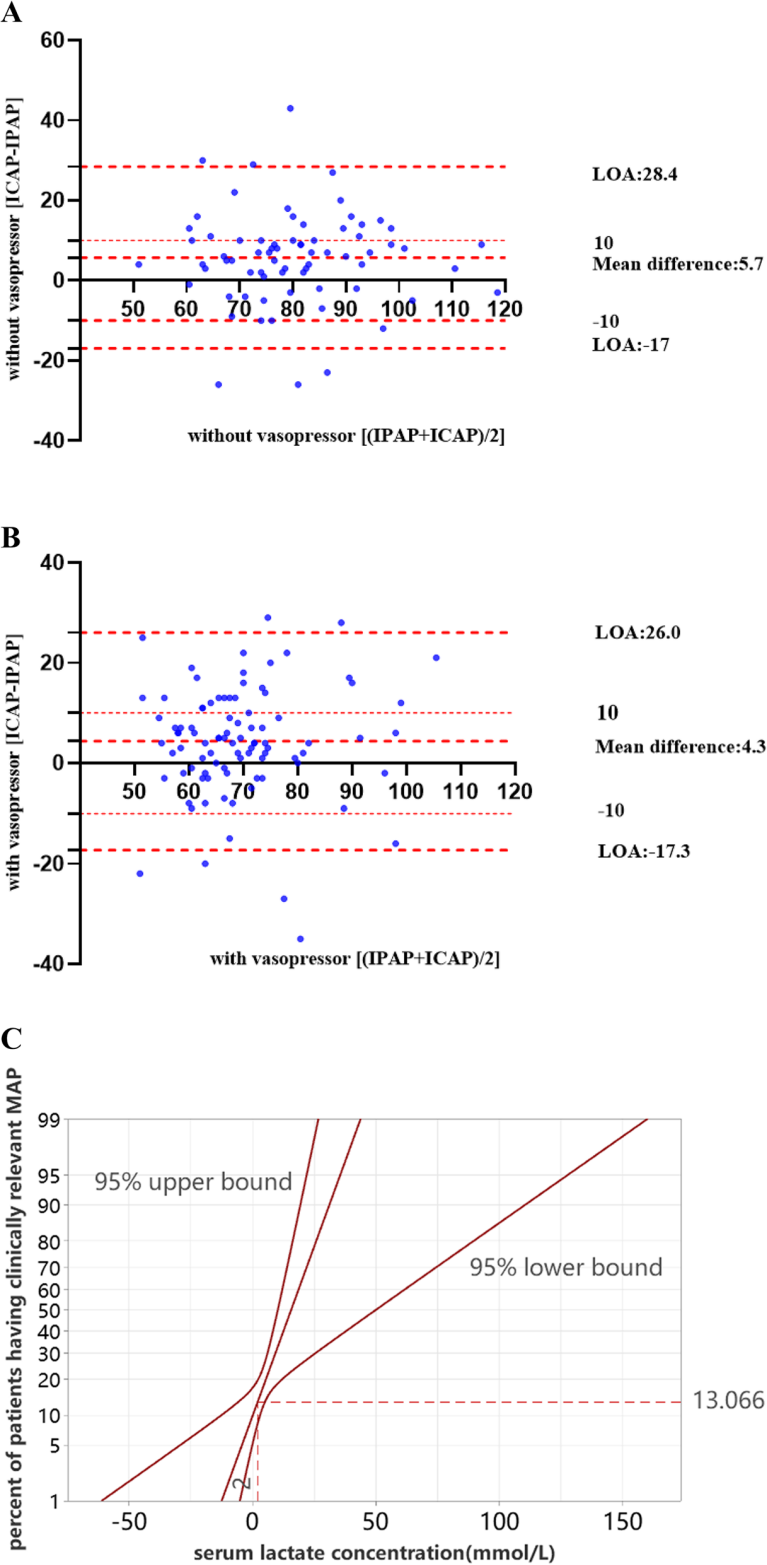
**A** Bland-Altman plot displaying blood pressure differences among patients undergoing IABP without vasopressors. IABP, intra-aortic balloon pumping; IPAP, invasive peripheral arterial pressure; ICAP, invasive intra-aorta pressure; LOA, limits of agreement. **B** Bland-Altman plot displaying blood pressure differences among patients undergoing IABP with vasopressors. IABP, intra-aortic balloon pumping; IPAP, invasive peripheral arterial pressure; ICAP, invasive intra-aorta pressure; LOA, limits of agreement. **C** Probit logit analysis showing probability of having a clinically significant discrepancy between ICAP and IPAP (Y-axis) and its association with serum lactate level. MAP, mean arterial pressure; ICAP, invasive central aortic pressure; IPAP, invasive peripheral arterial pressure

The Bland–Altman plots for patients with IABP (Fig. 2A, B) demonstrated an even distribution of the [ICAP-IPAP] discrepancy along the X-axis, indicating a uniform variance in the measurements obtained through the two modalities, irrespective of whether the patients were hypotensive or normotensive. Among patients not receiving vasopressors, the mean difference was 5.73 (SD 11.59), with 95% limits of agreement (LOA) ranging from -16.98 to 28.44; 8% (6/71) of the data points fell outside this range. For patients receiving vasopressors, the mean difference was 4.36 (SD 11.06) with 95% LOA between -17.31 and 26.03; 6% (6/91) of the data points were outside this range. The majority of the differences between ICAP and IPAP ([ICAP-IPAP] on the Y-axis) for this cohort were positive, indicating higher ICAP measurements compared to IPAP among patients undergoing IABP.

### Percentage of patients with clinically relevant MAP differences

In our study cohort, 17% (27/162) of the patients exhibited clinically relevant MAP differences (≥ 10 mmHg) that could necessitate alterations in clinical management. Among the patients requiring vasopressors, 20% (18/91) had clinically relevant MAP differences, in contrast to 13% (9/71) of those not requiring vasopressors (*p* = 0.229) (Table 2). This distribution implied that the requirement of vasopressors did not significantly impact the presence of clinically relevant MAP differences.

**Table 2:** Comparison of blood pressure between ICAP and IPAP monitoring modalities among patients undergoing IABP therapy with or without vasopressors

Variables	All patients (*n* = 162)	Without vasopressors (*n* = 71)	With vasopressors (*n* = 91)	*p* value
ICAP days, median [IQR]	5 [4–7]	5 [4–6.5]	6 [4–8]	0.124
IPAP days (days), median [IQR]	6 [4–8]	6 [4–8]	6 [3.5–9]	0.561
Type of vasopressors, n (%)				
Norepinephrine	12 (8)	0 (0)	12 (13)	NA
Epinephrine	13 (8)	0 (0)	13 (14)	NA
Dopamine	88 (54)	0 (0)	88 (97)	NA
Metaraminol	24 (30)	0 (0)	24 (26)	NA
HR, M (SD)	92 (21)	90 (18)	94 (23)	0.23
MAP as measured by ICAP (mmHg), M (SD)	77 (15)	83 (14)	72 (13)	< 0.001
MAP as measured by IPAP (mmHg), M (SD)	72 (14)	77 (15)	68 (12)	< 0.001
Difference of MAP between IPAP and ICAP measurements (mmHg), M (SD)	5 (11)	6 (12)	4 (11)	0.42
Patients with MAP difference 0–9 mmHg, n (%)	98 (61)	42 (59)	56 (62)	0.953
Patients with MAP difference 10–19 mmHg, n (%)	44 (27)	20 (28)	24 (26)	
Patients with MAP difference ≥ 20 mmHg, n (%)	20 (12)	9 (13)	11 (12)	
Number of patients MAP as measured by IPAP ≤ 69 mmHg, n (%)	75 (46)	17 (24)	58 (64)	< 0.001
Number of patients MAP as measured by ICAP ≤ 69 mmHg, n (%)	59 (36)	18 (25)	41 (45)	0.007
ICAP MAP > IPAP MAP Patients, n (%)	120 (73)	54 (76)	66 (72)	0.508
IPAP MAP > ICAP MAP Patients, n (%)	40 (25)	17 (24)	23 (26)	0.845
IPAP MAP = ICAP MAP Patients, n (%)	2 (1)	0 (0)	2 (2)	NA
Number of patients with a clinically significant discrepancy in MAP, n (%)	27 (17)	9 (13)	18 (20)	0.229
Complications of ICAP, n (%)	10 (6)	6 (8)	4 (4)	0.462
Complications of IPAP, n (%)	11 (7)	4 (6)	7 (8)	0.840

*IABP* intra-aortic balloon pump, *ICAP* invasive central aortic pressure, *IQR* interquartile range, *HR* heart rate, *M* mean, *SD* standard deviation, *IPAP* invasive peripheral arterial pressure, *MAP* mean arterial pressure, *NA* not applicable

### Predictors of clinically significant discrepancy between ICAP and IPAP in patients undergoing IABP

Multivariate logistic regression analysis showed that for each mmol/L increase in serum lactate, there was an associated 11% increase in the odds of observing a clinically relevant MAP difference between ICAP and IPAP measurements (OR, 1.14; 95% CI, 1.03–1.27; *p* = 0.013). Furthermore, patients aged 60 years or older had 13 times higher odds compared to those younger than 45 years (OR, 13.20; 95% CI, 1.50–115.51; *p* = 0.020). Conversely, patients with CHD were less likely to have clinically relevant MAP differences (OR, 0.34; 95% CI, 0.13–0.90; *p* = 0.031). The logistic regression model demonstrated a good fit (Hosmer–Lemeshow *p* = 0.65), and no collinearity was detected as the variance inflation factors for the final variables were below 10 (Table 3 and Supplemental file 2 for independent variable assignment methods).

**Table 3:** Results from forward stepwise multivariate logistic regression measuring association between clinical factors and the likelihood of clinically significant discrepancy between ICAP and IPAP measurements

Variables	OR	95% CI	*p* value	VIF
***Outcome: clinical relevance of the difference of ICAP and IPAP measurements***				
Serum lactate	1.14	1.03–1.27	0.013	1.03
Coronary heart disease	0.34	0.13–0.90	0.031	1.12
Age ≥ 60 years old	13.20	1.50–115.51	0.020	6.00
60 > Age ≥ 45 years old	6.20	0.7–54.38	0.099	5.81

*OR* odds ratio, *CI* confidence interval, *VIF* variance inflation factor, *ICAP* invasive central aortic pressure, *IPAP* invasive peripheral arterial pressure

Probit regression confirmed that serum lactate levels were associated with clinically relevant differences in MAP between ICAP and IPAP. The probit analysis showed that approximately 13% (95% CI, 9%-21%) of patients would present a clinically relevant MAP difference at a serum lactate level of 2 mmol/L (Fig. 2C). Therefore, arterial blood pressure monitoring could potentially influence clinical management in approximately one out of every 13 patients with serum lactate levels equal to 2 mmol/L.

### Percentage of patients exhibiting differences ≥ 10 mmHg between ICAP and IPAP measurements

Of the study population, 40% (64/162) demonstrated a MAP difference of 10 mmHg or greater between the two blood pressure measurement methods. Multivariate logistic regression analysis indicated that patients aged over 60 years were significantly more likely to have such MAP differences (OR, 9.78; 95% CI, 2.91–32.86; *p* < 0.01), whereas patients with CHD were less likely (OR, 0.38; 95% CI, 0.17–0.85; *p* = 0.02). The model did not exhibit collinearity (Hosmer- Lemeshow *p* = 0.13). The results are shown in Table 4.

**Table 4:** Results from forward stepwise multivariate logistic regression measuring association between clinical factors and the likelihood of clinically significant discrepancy between ICAP and IPAP measurements

Variables	OR	95% CI	*p* value	VIF
***Outcome: MAP difference* ≥ *10 mm Hg***				
Coronary heart disease	0.38	0.17–0.85	0.02	1.25
Age ≥ 60 years old	9.78	2.91–32.86	< 0.01	3.30
60 > Age ≥ 45 years old	4.55	1.41–14.64	0.011	2.99

*OR* odds ratio, *CI* confidence interval, *VIF* variance inflation factor, *MAP* mean arterial pressure, *ICAP* invasive central aortic pressure, *IPAP* invasive peripheral arterial pressure

## Discussion

Current guidelines advocate for maintaining a MAP of at least 70 mmHg to mitigate the risk of hypoperfusion [10]. However, the literature remains deficient in studies comparing different MAP monitoring techniques in CS patients who are undergoing IABP therapy. In our retrospective analysis, we observed a statistically significant discrepancy between the MAP values obtained via IPAP and ICAP. Notably, this discrepancy did not vary between patients receiving or not receiving vasopressors.

Our findings indicate that IPAP significantly underestimates MAP, with 17% (27/162) of the patients demonstrating a variance in MAP between ICAP and IPAP measurements. According to the Bland–Altman plot, the IPAP-derived MAP was consistently lower than that obtained through ICAP, irrespective of vasopressors administration. Consequently, in scenarios where IABP monitoring is infeasible due to sensor failure, transducer malfunctions, or console screen visibility issues, clinicians should remain vigilant to the possibility of underestimating blood pressure by up to 10 mmHg when using invasive MAP measurements as an alternative.

Furthermore, our study contradicts previous research regarding the influence of vasopressors on the discrepancy between ICAP and IPAP. Tran et al. suggested that vasopressors contributed to a 6.4-fold increase in clinical MAP compared to cases without vasopressors [25]. Contrary to this assertion, our analysis found that vasopressor usage did not have a statistically significant impact on this discrepancy. As vasopressors activate various receptors, they produce diverse hemodynamic effects on the central and peripheral vasculature. According to pharmacology, norepinephrine mainly stimulates α_1_ receptors, while dopamine acts on α, β, and dopamine receptors. The discrepancy in findings between our study and prior research may be attributed to differences in study populations and the fact that our study did not differentiate between various types of vasopressors. Moreover, a recent study by Kim et al. suggested that the severity of vasopressor usage plays a role [22]. Our cohort mainly consisted of patients with stable hemodynamics who were administered lower vasopressor doses, in contrast to Kim et al.’s cohort, which was primarily comprised of patients with septic shock receiving higher vasopressor doses. Future research should explore the associations between varying types and dosages of vasopressors and the discrepancies in MAP as measured by IPAP and ICAP.

The logistic regression analysis revealed a positive association between higher serum lactate levels, age ≥ 60 years, and the absence of CHD with an increased likelihood of a clinically relevant difference in MAP as measured by IPAP and ICAP. There is a scarcity of research investigating the factors associated with this clinically relevant difference in MAP among CS patients undergoing IABP therapy. In terms of serum lactate, our study corroborates the findings of Tran et al., where serum lactate levels were identified as a common independent risk factor for clinically relevant MAP differences in septic patients [25]. Moreover, Meaghan suggested that among 17 patients with serum lactate levels equal to or above 2 mmol/L, one might require a change in management owing to arterial blood pressure monitoring [13]. Additionally, a larger MAP discrepancy (greater than 10 mmHg) was noted in older patients without CHD. It is intriguing that CHD was linked to a lower likelihood of clinically relevant MAP differences. Research has indicated that SBP and DBP do not vary significantly between peripheral and central arteries in hypertensive patients with CHD [26, 27]. Patients with CHD experience an increase in arterial stiffness, leading to an elevation in pulse wave velocity. This, in turn, shortens the time required for the pressure wave to travel to and from the distal reflection point and the ascending aorta, causing an overlap of the reflected wave during the late systolic pressure of the central artery. Consequently, there is an increase in the systolic pressure of the left ventricle, which directly impacts coronary artery perfusion. Therefore, the severity of arteriosclerosis lesions in CHD patients plays a crucial role, resulting in only marginal clinical differences in MAP. Our results underscore the necessity of vigilant monitoring, particularly in patients over 60 years of age, exhibiting elevated serum lactate levels, and without CHD.

Additionally, we observed a 7% complication rate associated with IPAP (11/162), while it was 6% for ICAP (10/162). These findings align with previous reports, which state that the complication rate for patients undergoing IABP therapy ranges from 4.7% to 31.0%, with access site bleeding being the most prevalent complication [28]. Christenson suggested that protracted treatment duration is an independent risk factor for balloon-related complications [29]. Hence, expediting catheter removal and minimizing the duration of treatment are vital for reducing the risks of blood flow infections and other complications, thereby reduces the financial burden on patients.

Despite providing important insights into the differences between ICAP and IPAP measurements under various conditions and their potential impact on clinical management, this study has limitations. First, this was a retrospective study and data were derived from electronic medical records. It does not confirm causal relationships. Second, being a single-center study with a limited sample size, the findings may not be generalizable. Additionally, propensity score matching was not feasible, and the heterogeneity between patients requiring vasopressors and those who did not may induce bias due to different vasopressor mechanisms. Third, capturing only the three most recent blood pressure measurements post-IABP implantation might not reflect longitudinal trends. Other clinical factors not included in the current study might also influence the results, and the clinical relevance of these differences concerning patient outcomes remains unclear. Future multi-center randomized controlled trials with larger sample sizes, encompassing a broader range of variables and extended follow-up periods, are warranted to solidify the evidence.

## Conclusion

In conclusion, it is crucial to accurately monitor blood pressure for patients with CS undergoing IABP therapy. However, the consistency between IPAP and ICAP measurements is suboptimal. The current study revealed no statistically significant differences in MAP measurements between the groups treated with or without vasopressors. It is essential for physicians to comprehend the underlying technology and engage in dialogues with providers (e.g., vasopressor titrations). In managing CS patients undergoing IABP therapy, particular attention should be paid to individuals aged 60 years or above, exhibiting elevated serum lactate levels, and without a history of CHD, by judiciously adjusting vasopressor dosages and tailoring therapeutic regimens. Furthermore, this study highlight the importance of healthcare professionals employing peripheral invasive monitoring for patients receiving IABP therapy until additional research becomes available. It is vital to avoid premature withdrawal of vasopressors, which may result in insufficient organ perfusion, and to remain vigilant to the risks associated with arterial catheterization.

### Supplementary Information

**Additional file 1: Supplementary file 1.** Data extraction form.

**Additional file 2: Supplementary file 2.** List of independent variables that were included in the multivariate logistic regression that measured the association between clinical factors and clinically relevance of blood pressure monitoring of IABP patients.

## Data Availability

The data supporting the conclusions of this article are included within the article. Comprehensive aggregate data will be shared from the corresponding author upon request.

## Abbreviations

BMI: Body mass index
CCU: Coronary care unit
CHD: Coronary heart disease
CI: Confidence interval
CS: Cardiogenic shock
DBP: Diastolic blood pressure
ECMO: Extracorporeal membrane oxygenation
HTN: Hypertension
IABP: Intra-aortic balloon counterpulsation
ICAP: Invasive central aortic pressure
IMV: Invasive mechanical ventilation
IPAP: Invasive peripheral arterial pressure
IQR: Interquartile range
LAD: Left anterior descending
LCX: Left circumflex
LOA: Limits of agreement
LVEF: Left ventricular ejection fraction
M: Mean
MAP: Mean arterial pressure
NIBP: Non-invasive blood pressure
NIMV: Noninvasive mechanical ventilation
OR: Odds ratio
PAD: Peripheral arterial disease
RCA: Right coronary artery
SBP: Systolic blood pressure
SD: Standard deviation
