# Supplementary material for: Discrepancy between two invasive blood pressure measurements in patients receiving intra-aortic balloon pump therapy
Source: BMC Cardiovasc Disord. 2023 Sep 9;23:445. doi: 10.1186/s12872-023-03479-2 (PMC10493012; doi:10.1186/s12872-023-03479-2)
Supplement: Supplementary file 1 — Additional file 1: Supplementary file 1. Data extraction form. [file 12872_2023_3479_MOESM1_ESM.docx]

**Supplemental file 1**

| Data Extraction Form | | | | | | | | | |
| --- | --- | --- | --- | --- | --- | --- | --- | --- | --- |
| Patient number  Items | | 1 | 2 | 3 | 4 | 5 | 6 | 7 | 8 |
| Name | |  |  |  |  |  |  |  |  |
| Admission number | |  |  |  |  |  |  |  |  |
| **First Part** | |  |  |  |  |  |  |  |  |
| Age | |  |  |  |  |  |  |  |  |
| Sex | Male |  |  |  |  |  |  |  |  |
|  | Female |  |  |  |  |  |  |  |  |
| BMI | |  |  |  |  |  |  |  |  |
| Past histories | Diabetes |  |  |  |  |  |  |  |  |
|  | HTN |  |  |  |  |  |  |  |  |
|  | CHD |  |  |  |  |  |  |  |  |
|  | Kidney disease |  |  |  |  |  |  |  |  |
|  | PAD |  |  |  |  |  |  |  |  |
| Location of arterial catheter | Radial |  |  |  |  |  |  |  |  |
|  | Brachial |  |  |  |  |  |  |  |  |
| Diagnoses | |  |  |  |  |  |  |  |  |
| Mechanical ventilation | None |  |  |  |  |  |  |  |  |
|  | IMV |  |  |  |  |  |  |  |  |
|  | NIMV |  |  |  |  |  |  |  |  |
| Infarct culprit artery | LAD |  |  |  |  |  |  |  |  |
|  | RCA |  |  |  |  |  |  |  |  |
|  | LCX |  |  |  |  |  |  |  |  |
| Serum lactate |  |  |  |  |  |  |  |  |  |
| LVEF | Discharge home |  |  |  |  |  |  |  |  |
| Hospital disposition | Dead/Hospice |  |  |  |  |  |  |  |  |
| **Second Part** | | | | | | | | | |
| ICAP days |  |  |  |  |  |  |  |  |  |
| IPAP days |  |  |  |  |  |  |  |  |  |
| Type of vasopressor | Norepinephrine |  |  |  |  |  |  |  |  |
|  | Epinephrine |  |  |  |  |  |  |  |  |
|  | Dopamine |  |  |  |  |  |  |  |  |
|  | Metaraminol |  |  |  |  |  |  |  |  |
| HR |  |  |  |  |  |  |  |  |  |
| MAP as measured by ICAP |  |  |  |  |  |  |  |  |  |
| MAP as measured by IPAP |  |  |  |  |  |  |  |  |  |
| Difference of MAP between IPAP and ICAP measurements |  |  |  |  |  |  |  |  |  |
| Patients with MAP difference 0–9 mmHg |  |  |  |  |  |  |  |  |  |
| Patients with MAP difference 10–19 mmHg |  |  |  |  |  |  |  |  |  |
| Patients with MAP difference ≥20mmHg |  |  |  |  |  |  |  |  |  |
| Number of patients MAP as measured by IPAP≤ 69 mm Hg |  |  |  |  |  |  |  |  |  |
| Number of patients with MAP as measured by ICAP ≤ 69 mm Hg |  |  |  |  |  |  |  |  |  |
| ICAP MAP>IPAP MAP Patients |  |  |  |  |  |  |  |  |  |
| IPAP MAP>ICAP MAP Patients |  |  |  |  |  |  |  |  |  |
| IPAP MAP=ICAP MAP Patients |  |  |  |  |  |  |  |  |  |
| Number of patients with a clinically significant discrepancy in MAP |  |  |  |  |  |  |  |  |  |
| Complications of ICAP |  |  |  |  |  |  |  |  |  |
| Complications of IPAP |  |  |  |  |  |  |  |  |  |
| BMI, body mass index; HTN, hypertension; CHD, coronary heart disease; PAD, peripheral arterial disease; IMV, invasive mechanical ventilation; NIMV, noninvasive mechanical ventilation; LAD, left anterior descending; RCA, right coronary artery; LCX, left circumflex; LVEF, left ventricular ejection fraction. ICAP, invasive intra-aorta pressure; IQR, interquartile range; HR, heart rate; IPAP, invasive peripheral arterial pressure; MAP, mean arterial pressure. | | | | | | | | | |
