# Supplementary material for: Discrepancy between two invasive blood pressure measurements in patients receiving intra-aortic balloon pump therapy
Source: BMC Cardiovasc Disord. 2023 Sep 9;23:445. doi: 10.1186/s12872-023-03479-2 (PMC10493012; doi:10.1186/s12872-023-03479-2)
Supplement: Supplementary file 2 — Additional file 2: Supplementary file 2. List of independent variables that were included in the multivariate logistic regression that measured the association between clinical factors and clinically relevance of blood pressure monitoring of IABP patients. [file 12872_2023_3479_MOESM2_ESM.docx]

**Supplemental file 2.** List of independent variables that were included in the multivariate logistic regression that measured the association between clinical factors and clinically relevance of blood pressure monitoring of IABP patients.

| **Variables** |
| --- |
| ***Continuous variables*** |
| Body Mass Index -each unit |
| Serum Lactate level - each mmol/L |
| LVEF-each unit |
| ***Categorical variables*** |
| Gender (Male =1; Female =0) |
| Age (≥60=2; ≥45=1; ≥14=0) |
| Location of arterial catheter (Radial = 1; Brachial = 0) |
| Past medical of Diabetes (Yes = 1; No =0)  Past medical history of Coronary Heart Disease (IMV =2; NIMV =1; NONE=0) |
| Past medical history of Hypertension (Yes=1; No=0) |
| Past medical of Peripheral Artery Disease (Yes=1; No=1) |
| Past medical history of Any Kidney Disease (Yes=1; No=0) |
| Receiving Any Vasopressors (Yes = 1; No =0)* |
| *Vasopressor includes Norepinephrine, Epinephrine, dopamine and metaraminol; mmol/L, millimoles per liter; IABP=Intra-aortic balloon pump; LVEF=Left ventricular ejection fraction; IMV, invasive mechanical ventilation; NIMV, non-invasive mechanical ventilation. |
